# Effect Morphology and Surface Treatment of the Abutments of Dental Implants on the Dimension and Health of Peri-Implant Biological Space

**DOI:** 10.3390/ma15134422

**Published:** 2022-06-22

**Authors:** Ibrahim Dib-Zaitum, Yasmina Guadilla-González, Javier Flores-Fraile, Juan Dib-Zakkour, Lorena Benito-Garzón, Javier Montero

**Affiliations:** 1 Department of Surgery, Faculty of Medicine, University of Salamanca, 37007 Salamanca, Spain; ibrahimdib@usal.es (I.D.-Z.); yguadilla@usal.es (Y.G.-G.); juandib@usal.es (J.D.-Z.); javimont@usal.es (J.M.); 2 Department of Human Anatomy and Histology, Faculty of Medicine, University of Salamanca, 37007 Salamanca, Spain; lorenabenito@usal.es

**Keywords:** dental abutment, peri-implantitis, titanium surface treatment

## Abstract

Statement of the problem: The gingival configuration around implant abutments is of paramount importance for preserving the underlying marginal bone, and hence for the long-term success of dental implants. Objective: The objective was to study, clinically and histologically, the effects of the change in the morphology of abutments connected to the endosseous implant, and of their surface treatment. In particular, the objective was to ascertain the effect of changing the shape of the transepithelial pillar and the treatment of its surface on the dimensions, quality and health of the components of the peri-implant biological space, such as the dimensions of the epithelial and connective tissues of the biological space, the concentration of inflammatory cells and the density of collagen fibers. Methods: A clinical trial of 10 patients with a totally edentulous maxilla, who had four implants (IPX4010_GALIMPLANT^®^, Sarria, Spain) inserted in the area of the first and second molars on both sides with computer-guided implant surgery, was conducted with the final purpose of assessing the quality of the peri-implant soft tissue attachment around the transepithelial abutments which were employed (aesthetic machined (RM), aesthetic anodized (RA), slim machined (SM) and slim anodized (SA)). At 8 weeks and following the collection of the samples (removal of the implant-abutment assembly with its surrounding hard and soft tissue) and their processing for subsequent histological and histomorphometric analysis in order to study the dimensions, quality and health of the peri-implant soft tissue area, the variables previously mentioned were determined according to the aims of the study. By using appropriate diameter trephine in order to obtain a useful fringe of soft tissue around the transepithelial pillars, ANOVA and chi-square tests were performed. Results: The SPSS statistical analysis ANOVA results revealed that the machined slim abutments have a better performance considering the variables analyzed with epithelial and connective attachment heights of 1.52 mm and 2.3 mm, respectively, and that connective density (density of collagen fibers) was high at 85.7% of the sample size affected by the design for the slim abutments and 92.9% of the high-density sample size affected by the surface treatment for the machined surface. Conclusions: All variables studied, despite the small sample size, showed the superiority of the slim machined abutment among the four groups.

## 1. Introduction

Dental implants are a very common treatment option for both partially and fully edentulous patients due to their long-term success (Buser et al., 2012; Gotfredsen, 2012) [1,2]. However, one of their most feared complications is peri-implantitis, which involves a loss of peri-implant bone tissue due to bacterial invasion of peri-implant tissues as a result of an imbalance between bacterial quantity/quality and the host’s defensive capabilities [3]. Peri-implant tissues have a lower capacity for soft tissue sealing (epithelial and connective) than the original tooth [4]. This protective band of connective tissue and epithelium is known as the biological space, with dimensions ranging from 1.2 to 2.0 mm in height for epithelial tissue and 1.0 to 1.5 mm in height for connective tissue [5]. These dimensions correlate to the degree of bone remodeling that occurs after the connection of the abutment [6,7,8].

The effect of various morphological characteristics, surface treatments and manufacturing materials on early peri-implant bone loss has been studied with the aim of promoting a good epithelial-connective seal which will protect the peri-implant marginal bone [9,10].

Weilander [9] found that a new design of abutments with concave microgrooves did not present significant differences compared to convex microgrooves. Iglhaut, Becker and Mihativic, 2013, and Kim et al., 2010 [10], studied the variation of biological width with regards to the surface treatment of the abutments, observing a greater epithelial thickness on smooth machined titanium abutments (2.9 ± 0.4 mm) compared to rough surfaces (1.4–0.3 mm), but found no statistically significant differences in the morphology of the abutments with respect to the dimensions of the laser microgrooved margins. Studies on animals have shown an increased density of connective tissue attachment to abutments with microgrooved surfaces, with decreased alveolar bone loss (Rompen, 2012) [11].

In a study performed on dogs by Berglund et al., the morphogenesis of mucosal attachment occurred within 8 weeks [12]. This study found a considerable presence of inflammatory infiltrations caused by bacterial colonization on the abutments, causing loss of both epithelial and connective connections, and ending with loss of the peri-implant bone.

The quality of the oral mucosa as the first barrier when protecting the peri-implant bone [3] is the reason that most research is directed towards stabilizing the properties and health of the soft tissue, and towards the long-term adhesion of soft tissue to the prosthetic components [13]. The biological interaction between the properties of the abutment and the surrounding soft tissue has a great influence on adhesion between the two [14,15]. According to Canullo, 2016, fibroblast adhesion to the abutment surface is mediated by extracellular matrices (fibronectin, vitronectin, collagen, laminin or fibrin) [16,17], so the characteristics of the abutment surface must facilitate such interactions while also bearing in mind the high probability of bacterial contamination [18].

Recent studies have shown the topographic modification of the abutment surface with the formation of micro furrows, thus expanding the surface, and its roughness promotes the appearance of perpendicular collagenous connections [19,20], because cell adhesion on the abutment surface occurs through the formation of hemidesmosomes similar to those in natural teeth [21,22].

In view of the above, the influence of both morphology and surface treatments on the formation of the peri-implant soft tissue seal is striking [23]. Singh A.V. notes that surface topography has a greater influence than surface treatment with regards to bacterial adhesion [24].

Chrcanovic BR, 2014, considers that the hydrophilic and hydrophobic properties of the abutment surface are crucial in their influence on bacterial adhesion [25].

According to Mishra [26], the hydrophilic surface of the abutments minimizes the absorption of bacterial proteins and biofilm formation, while Canullo et al. [27] attach greater importance to the technique used in the surface treatment.

In recent years, some studies have been reported employing nanotube structures as micro-nano electrode interfaces to detect cell activities in in vivo and in vitro environments. We believe nanotubes can play a key role in becoming such an interface due to their inherent biocompatibility leading to great cell proliferation, adhesion and mineralization [23,24,25,26].

The prosthetic attachments used have been previously anodized, converting the geometry of their surface into nanotubes, which favor gingival biology. Anodizing consists of a surface treatment that is carried out on aluminum to form an oxide layer of that material. The creation of this layer is controlled and is carried out through an electrolytic process. During this process, a direct current is passed through the aluminum surface, which ends up behaving like an anode in an acid medium. The surface layer that is generated with anodizing is superficial, protective, thin and passive microporous [27].

The aim of our study is to evaluate the response of the peri-implant soft tissue in terms of dimensions and quality against the change in the topography and the treatment of the surface of the transepithelial abutments to histomorphometrically evaluate the response of peri-implant soft tissue to various designs of transgingival abutment (topography), with or without surface treatment; that is, to study the effect of the shape and its surface treatment on fibrous neoformation (dimensions of the biological space and density of the collagen fibers in the transgingival area).

The hypothesis of our study is that the use of slim abutments with or without surface treatment will improve the quality of the biological width.

## 2. Materials and Methods

All participants expressed a desire to wear an upper overdenture. All were adequately informed on the surgical procedures, first through a personal interview and then at the time of signing the informed consent previously approved by the Bioethics Committee of the University of Salamanca 393, June 2019.

The present study was designed following the principles of the 2008 Declaration of Helsinki concerning experiments involving humans. This clinical trial is registered under number NCT05284461 and the full protocol may be accessed at www.clinicaltrials.gov (accessed on 20 April 2022).

### 2.1. Patients and Subgroups

The inclusion criteria were completely edentulous patients with over 10 years of use of conventional complete dentures with enough residual bone in the molar area to place two implants per hemiarch.

The exclusion criteria were suffering from temporomandibular pathology and present evidence of any systemic or psychological pathology that would contraindicate treatment with implants.

The patient’s age did not form any condition for inclusion or exclusion; the only criterion applied in this situation was to have been in a situation of total edentulousness rehabilitated with a muco-supported complete prosthesis for at least 10 years.

In relation to the sex and smoking status of the patients selected to be included in the trial, the two variants were not taken into account, despite the fact that data were recorded and included in the statistical study and are provided below (Table 1).

This clinical trial was designed to assess the histological and histomorphometric characteristics of four different transepithelial abutments for which 40 implants were placed and, after two months, removed together with the surrounding tissues for examination. The tissue collected at the time of collecting the samples was both the hard and soft tissue that surrounds both the implant and the abutment of each and every one of the samples.

A selection of the patients examined with conditions indicated to be operated on by guided surgery was taken from the sample by computed tomography scan. From the 12 pre-selected patients, only 10 had enough bone to accommodate the 4 posterior implants for this study. Prior to treatment, patients were adequately informed of the surgical procedures, first verbally and then by signing the informed consent form proposed by the academic institution. All clinical procedures were carried out at the USAL Dental Clinic (Salamanca, Spain) and by the same doctor.

**Figure 1:**
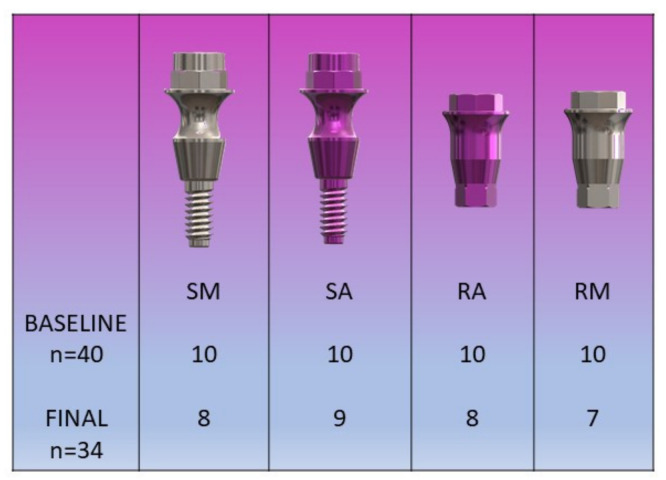
Group distribution. Abutment design: SM (slim machined), SA (slim anodized), RA (regular anodized), RM (regular machined).

The sample consisted of 10 patients who received 60 implants (IPX-4010_GALIMPLANT^®^, Sarria, Spain) in the atrophic maxilla by guided surgery. Of the 60 implants, 40 were studied, divided into 4 groups according to the Galimplant Dental System (Sarria, Spain) transepithelial employed in each implant (Figure 1).

RA (n = 10): Regular anodized. Reduced platform, anodized surface with a roughness of 450 microns.

RM (n = 10): Regular machined. Reduced platform, machined surface.

SM (n = 10): Slim machined. Non-reduced platform diameter, concave in the center, machined surface.

SA (n = 10) Slim anodized. Non-reduced platform diameter, concave in the center, anodized surface with a roughness of 450 microns.

### 2.2. Protocol

From the information in the DICOM format obtained by the computed tomography, and from the scan of the intraoral mucosa (3Shape TRIOS^®^ Model S1P) with the program “CO-diagnostic Straumann Dental Wings”, surgical stents were created for each of the six implants, one anterior and two posterior implants per hemiarch. The 4 posterior implants were the subjects of this study, while the 2 anterior implants will support the overdenture in the future.

Once the surgical stent was in place, the clinical protocol began by removing the mucous plug with a circular scalpel of equal outer diameter to the implant, and the drilling sequence indicated by the guided surgery protocol was followed. The 6 implants (IPX-4010_GALIMPLANT^®^, Sarria, Spain) were placed in the upper arch at a juxta-osseous level. Implants were always placed in the same order: anterior to posterior, right to left.

The two anterior implants were allowed to heal with a standard healing abutment. In the 4 posterior implants, the transepithelial abutments being studied were screwed with a torque of 30 Nw. The 4 abutments intended for each patient were kept in a black bag. As the implants were placed, a box containing an abutment was selected blindly and the abutment placed in each implant. Broad-spectrum antibiotics and anti-inflammatories were prescribed for 5 days, and local application of Bexident Post ISDIN gel three times a day for a week was recommended. During the same appointment, the removable prostheses were adapted to the new conditions through the use of tissue conditioners, as a form of provisional rehabilitation.

Patients were re-examined one week and one month after surgery; clinical measurements and radiological images (orthopantomography) of the patients were obtained on the day of the intervention and one month and two months after surgery. Biopsies, including the implant and the abutment with the surrounding soft and hard tissues, were taken two months after implant placement with the help of the modified surgical stent, a circular scalpel and a Galimplant ^®^ 5 mm inner diameter trephine burr. The samples were placed in a 10% formaldehyde buffered solution, marked by the same member of the team and labelled with a code known only to him. These samples were then deposited in the USAL histology service.

Two months later, the 2 anterior implants were connected with a Galimplant^®^ locator-like accessory and the overdentures were refitted.

### 2.3. Variables/Data Acquisition

After removing the mucous plug, gingival mesial thickness was measured with a CP15 periodontal probe, from the bone level to the mucosal surface.

**Figure 2:**
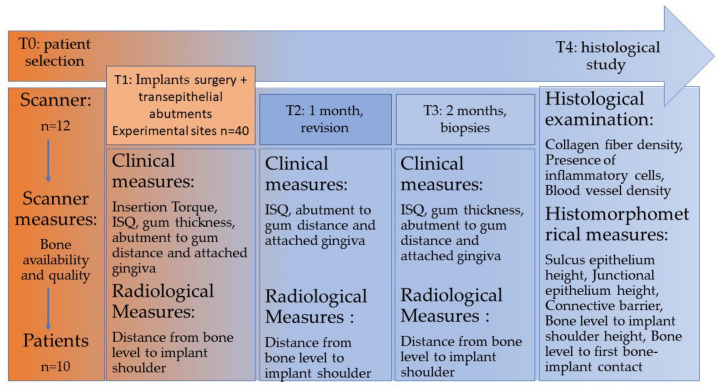
Timeline of stages performed.

At the end of the surgery, various clinical measurements were taken: implant insertion torque, implant stability quotient (ISQ, Osstell Mentor^®^ Instrument, Bürmoos Austria), buccal distance from the gingiva to the most coronal part of the abutment (CP15 probe) and buccal attached gingiva (CP15 probe). A standardized orthopantomography was taken to measure the distance from the bone level to the shoulder of each implant. During the one-month clinical review, the following measurements were again taken in the same standardized way: ISQ, distance from the gingiva to the most coronal part of the abutment and attached gingiva. The radiograph was taken again to measure the distance from the implant shoulder to bone level. At two months, ISQ, gingival mesial thickness, distance from the gingiva to the most coronal part of the abutment, attached gingiva and radiological measurements were performed again in the same way (Figure 2).

### 2.4. Histological Processing

The samples were introduced in Eppendorf tubes submerged in formalin reduced to 10% and kept at 4° of refrigeration until their processing described below. The samples containing the implants were gradually dehydrated in ethanol solutions with increasing concentrations from 70% to 100%. After dehydration, the samples were embedded in methyl methacrylate resin as an undecalcified block. The samples were cut with a low-speed microtome blade (Isomet, Bueher^®^ lake bluff, Dusseldorf, Germany), parallel to the implant axis, to obtain central sections. Sections were directly stained with Stevenel’s Blue and van Gieson’s picrofuchsin, which stain mineralized bone in red and non-mineralized matrix in blue [28].

**Figure 3:**
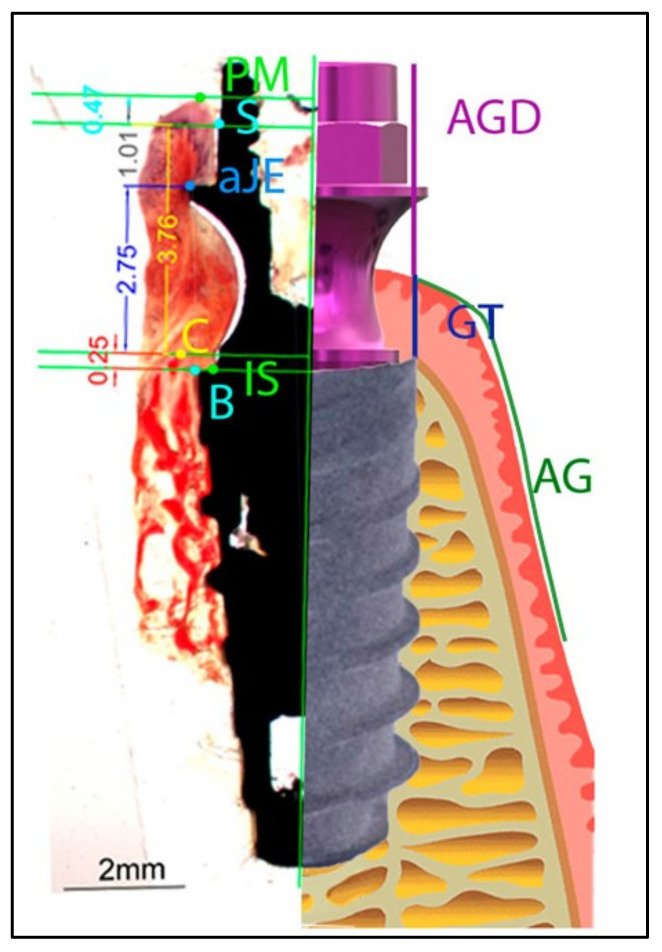
Diagram illustrating the landmarks for histomorphometric evaluation and the clinical measures: IS, implant shoulder; B, most coronal bone-to-implant contact location; C, the top of the alveolar crest; aJE, the apical border of the junctional epithelium; PM, the top of the margin of the peri-implant mucosa; AG, attached gingiva; GT, gum thickness; AGD, abutment to gum distance.

Histological examination was performed with a Nikon Eclipse 90i microscope equipped with a Nikon Sight DS-SMc digital camera (Nikon Instruments Inc., Melville, NY, USA). Photographs of the stained histological sections were taken with a Zeiss KL 1500 electronic loupe coupled to a Nikon DXm 1200 digital camera, giving a complete image of the implant and surrounding tissues. Histomorphometric measurements were taken on three slices per sample (Autodesk_AutoCAD 2019) with the help of a Nikon Eclipse 90i microscope to distinguish the tissues correctly. Each slice was divided into two parts along the implant axis and thus measurements were made on both sides. (Figure 3).

### 2.5. Acquisition of Histological and Histomorphometric Variables/Data

Once strategic points were established on the loupe photograph, the various distances between these points were measured for histomorphometric references: the height of the epithelial sulcus, connective barrier, height of the junctional epithelium, distance from bone level to the implant shoulder and height from bone level to the first bone-implant point of contact. (Figure 3).

Histological parameters (collagen fiber density, the presence of inflammatory cells and blood vessel density) were measured subjectively, establishing a four-tier system (absent, mild/low, moderate, notable/high) to objectify the quantity or density in each sample.

The analysis of variance with post hoc Bonferroni correction was used to compare quantitative variables among groups after checking the normal distribution of such variables. Chi-square tests were used to compare nominal variables across subgroups. Statistical analysis was performed with SPSS statistics (IBM, Armonk, NY, USA). The significance level (α) was set at 0.05.

## 3. Results

Table 2 shows the result of the fractional analysis of the effect of morphology and treatment of the abutment surface on the characteristics studied among the four groups of abutments: slim machined (SM), slim anodized (SA), regular machined (RM) and regular anodized (RA).

With regards to the depth of the peri-implant sulcus, the depth was significantly smaller around the 0.4 mm slim abutments compared to the 0.8 mm. The abutment morphology seemed to be more significant than the surface treatment on the gingival tissue configuration.

Table 3 compares collagen fiber density and the vascularization quantity between both subgroups within the design and surface groups. Despite there not being significant differences among the subgroups, the anodized abutments tended to be in contact with a denser connective tissue (Chi^2^ = 3.15; *p* = 0.08). Additionally, higher vascularization was observed within the slim transmucosal abutment in comparison with the so-called aesthetic abutment, although this difference was not statistically different (Chi^2^ = 1.38; *p* = 0.24).

As can be seen in Table 4, there were no significant differences regarding the concentration of inflammatory cells in the peri-implant soft tissues.

The statistical results of the above variables can be presented as graphs of the variation among groups, with each graph showing three times: T2 immediately after implantation, T3 at one month after implantation and T4 at two months after implantation.

Table 5 presents the variation of the ISQ among the three times. Positive response from the anodized slim abutments, as opposed to the other abutments, was observed after 2 months of implantation. This positive result can be initially awarded with validity in the short term.

Development and change in the distance between the shoulder of the abutment and the bone crest from the moment the implants were inserted until two months post-surgery were observed. Observed values of the slim anodized (SA) and aesthetic machined (RM) abutments were almost equal. When the reduced sample size and limited time elapsed are taken into consideration, we can say that with regards to this variable (distance from the abutment shoulder to the bone crest), there was no difference among the abutment groups (Table 6).

The width and development of the attached keratinized gingival over the two-month healing period from the moment of implantation to when samples were collected is shown in Table 7.

Slim machined (SM) abutments showed a better response with regards to the remodeling of the attached gingiva, without significant differences among the four groups (Table 7).

Table 8 shows the change in height of the peri-implant sulcus throughout the two-month healing period. A significant development of the peri-implant groove was observed around the aesthetic machined (RM) abutments, followed by the slim anodized (SA).

The magnitude of bone remodeling around the four abutment groups after 8 weeks of implantation showed a notable maintenance of the bone level in the group of slim machined (SM) abutments followed by the aesthetic anodized (RA) multi-position abutments with no significant difference between the four groups.

It shows the effect of abutment design and surface treatment on the following variables: the biological width, depth of the peri-implant sulcus, height of the epithelium and height of the connective tissue.

With regards to biological width, both designs on both surfaces were shown to have an approximately similar effect. Regular aesthetic abutments led to a greater depth of the peri-implant sulcus than slim abutments, with the effects of surface treatment being similar.

A greater height of the epithelium within the sulcus was observed in the group of anodized aesthetic abutments than in the other designs and surfaces.

With regards to the height of connective tissue within the peri-implant sulcus, both morphological designs were shown to have a similar effect, while regarding surface treatment, greater connective tissue height was observed in the machined abutments.

## 4. Discussion

The most influential characteristics associated with prosthetic abutments are morphology and surface treatment and, therefore, a large part of the research has focused on these two fields, as well as on the materials employed during their manufacture [28].

According to the traditional hypothesis, an increase in the roughness of the prosthetic abutment surface facilitates the formation of biofilm, a negative situation which influences clinical periodontal parameters [29].

Various authors support the hypothesis that peri-implant soft tissue connections provide better results with a moderately rough surface. In reality, soft tissue integration would, in the short term, be better with a roughened abutment surface [30,31]. This property becomes less efficient in the long term due to intense biofilm growth. When compared to the results of our study, this attributes greater importance to the design of the abutment (slim) and, secondly, smooth machined abutments gave better results with regards to peri-implant soft tissue attachment.

Hall et al. [32] analyzed the surface of anodized titanium, revealing the existence of antimicrobial properties which reduced microbial adherence to the abutments; this was in contrast to the results of our study, where we found better attachment at both the epithelial and connective levels in the non-anodized smooth titanium surface with an epithelium height on the smooth machined surface of 1.52 mm and a connective tissue height of 2.3 mm, while on the anodized surface, epithelium and connective heights were 2.02 mm and 1.74 mm, respectively.

Göthberg et al. [33] and Raes et al. [34] used an oxidized surface (Ti unite). Schwarz et al. [35] modified the surface of an abutment, applying a collar with a height of 0.7 mm and forming it with microgrooves, and compared it to a smooth titanium surface, finding no significant difference between the groups. Garcia et al. [36] and Canullo et al. [27,28] modified the titanium surface with argon plasma. In this type of treatment, it should be noted that the process does not modify the topography of the titanium but instead causes activation at both the atomic and molecular levels, thus increasing its wetting ability and preserving the surface integrity [37]. Such activation has been shown to accelerate the proliferation of peri-implant soft tissue cells [38]. Mehl et al. [39], in a study on abutments made from various materials (titanium, zirconium and lithium disilicate), stated that there is no significant difference in the anatomy of the soft tissue, except for a greater length of the epithelium in the titanium abutments when compared to zirconium.

Chien et al. [40] found a higher density of connective tissue arranged perpendicular to microgrooved abutments than in the machined titanium control group, where they found a lower density of collagen fibers, arranged parallel to the surface of the abutment and accompanied by apical migration of the epithelium. This situation is contrary to the results of our study, which found higher connective tissue density on the slim machined (SM) abutments. With these results in mind, we may conclude that the influencing factor is the morphology of the abutment rather than its surface treatment. Neiva et al. [41] and Nevins et al. [42] found similar results in laser-treated grooved abutments.

In 2015 [43], a trend was reported of an increasing percentage of collagen fibers on the surface of a modified abutment with a machined collar.

Secondly, authors could not demonstrate a difference in either quality or quantity between the surface of smooth, machined, thermally treated titanium abutments and abutments with a rough surface, concluding that peri-implant soft tissue attachment is unaffected by surface roughness. This result coincides with the results of our study, where a higher quality of soft-tissue attachment was found on a machined surface with another topography (slim) [6]. Teng et al. [44], in their study on polydopamine anodized and machined abutments, found no significant differences with regards to peri-implant connections.

The use of non-isotropically modified surfaces, such as micro-grooved abutments, is associated with a more coronal position in terms of peri-implant biological space, with a considerable density of connective collagen fibers oriented perpendicular to the abutment surface [34,36,40]. As for the shape of the abutments, the reduced platform has for the moment been shown to play an important role in the stability of the supra-crestal attachment of the peri-implant soft tissue, while concave transmucosal profiles have been proposed to improve the stability of these attachments, where a higher density of connective tissue has been observed [10]. These results are in agreement with our study, which found a high density of connective tissue in the slim (concave) abutments in 87.5% of the samples analyzed.

In a study on the effect of abutment surface treatment, an analysis of local levels of angiogenesis and osteoblastogenesis markers in peri-implant fluid during the initial phase showed that the surface treatment employed was related to the levels of the aforementioned markers [45].

Others authors observed an increase in the proliferation of osteoblasts and bacterial reduction on a surface treated with TiO_2_, with a positive effect on bone regeneration and vascular neoformation [46].

A meta-analysis determined that peri-implant soft tissue is not affected by the treatment of the abutment surface in the short term, a condition then reversed in the long term, making the technique chosen for surface treatment important [27]. The conclusions of Canullo L. are in agreement with the results of our study, where a notable positive change was observed in the connective tissue of the peri-implant sulcus after 8 weeks following the insertion of the abutment.

In another study, TiO_2_ received primary attention due to its high corrosion resistance, biocompatibility, inertness, low gravity and lack magnetism, which gives it high proliferation, adhesion and mineralization. Additionally, the authors stated that modifying the surface of TiO_2_ by grooves with a depth of 15–50 nanometers improves the above qualities [47]. In the year 2009, in an in vivo study, it was shown that the multi-layer structure of nanotubes on a TiO_2_ surface favors the adhesion of bone mesenchymal cells and osteoblasts compared to an untreated TiO_2_ surface [48,49].

In an in vitro study, it was shown that the ideal thickness of the TiO_2_ surface topography is 30–40 nm, a sufficient thickness to increase the cell transport load [50], whereas J.A. Sorkin, 2014, proved in vivo that the required thickness of the surface tubes could be reduced almost by half, thus improving conductivity and increasing surface area [51]. Sanz Martín, 2017, on the subject of surface topography, stated that there is no significant difference with regards to bleeding at the time of probing when different abutment topologies are considered. On the other hand, as for abutment material, a significant difference was observed, favoring zirconium abutments compared to titanium ones [52].

Regarding the degree of bacterial accumulation, Iglhaut et al., 2014, stated that bacterial colonization on the abutment surface and the implant–abutment interface increases inflammatory infiltration, minimizing epithelial connections and in turn affecting bone level [53], a statement that agrees with the results of Rompen’s in vivo study in 2012, which stated that contamination of the abutment surface has a negative effect on soft tissue integration [54].

In a study on abutments, it was stated that soft tissue attachment is influenced by the degree of surface roughness and the method by which it is created, with potential benefits on its integrity [55]. On the other hand, Iglhaut and Becker, in their 2013 study on dogs, determined that the dimensions of the biological space are almost identical across all abutment surfaces. The only variation was in the height of the epithelial connection (machined titanium, 2.9 mm versus rough, 1.4–1.6 mm) [10]. These results agree with those obtained in our clinical trial, with dimensions of 2.02 mm for the slim anodized (SA) abutments with a surface treatment to create a rough surface, whereas the machined aesthetic abutments in our study had a height of 1.52 mm, results which contrast with those obtained by Iglhaut and Becker [10].

In a study on dogs, the authors found that abutments with micro-grooves are associated with increased connective attachment height and less bone loss [56]. Our study, in comparison, found higher connective tissue height with the machined surface, 2.3 mm, whereas the tissue height on the anodized surface was 1.74 mm. The effect of abutment morphology must be taken into consideration, as it is a factor of great influence on the height of the biological space, especially in our study.

In vivo studies on humans compared conventional straight abutments with those treated with micro-grooves at the implant shoulder, obtaining greater soft tissue height with better marginal sealing without a noticeable effect on the bone [9,57].

Teughels et al., 2006, [29] in their study on humans, showed that the topography of the abutment has no effect on bleeding during probing. No change was observed at the bone level either, despite the hypothesis that the increased roughness of the abutment surface accumulates more plaque with greater inflammation and bleeding [29]. Comparing the results of Teughels et al. [29] with ours, and taking into account the hypothesis that roughness increases plaque and local inflammation, this study agrees with our results: that smooth machined abutments showed a lower concentration of inflammatory cells of 21.4% in the samples studied, while the anodized abutments had a concentration of 37.5% in the samples. On the other hand, abutment morphology returned positive results favoring the new slim design.

In a 2015 study on humans on the effect of plaque accumulation and peri-implant inflammatory infiltration with regards to abutment material, it was stated that zirconium showed less accumulation of bacterial plaque with a greater proliferation of fibroblasts when compared to titanium [58]. In addition, the combination of the treatment of the prosthetic accessories with the use of chlorhexidine can improve the biological behavior of the tissues, favoring the peri-implant gingival structure [59].

With regards to abutment dimensions, an in vivo study on the effect of abutment height and marginal bone loss (MBL) concluded that abutment height is related to MBL, but not linearly, and therefore MBL is greater in the first 6 months of loading on the implant and decreases at 12 months, concluding that the MBL ratio is greater with abutments under 2 mm high [60]; whereas Qian et al., 2012, [60] in an in vivo study, stated that the higher the abutment height, the greater the probability of pathogen retention, leading to the increased depth of the peri-implant sulcus and to soft tissue inflammation [61]. Vervacke et al., 2014, [61] in an in vivo study on humans, observed an increased MBL with short abutments due to the initial pressure from the thin mucosa [62].

On the shape of the abutment, in a systematic review on the relationship of keratinized mucosa (KM) to abutment morphology, it was stated that concave abutments favor the creation of KM compared to convex abutments (2.1 mm vs. 1.3 mm, respectively) [63]. These results agree with those of our study, where we found greater evolution of the keratinized gingiva accompanying the concave slim abutments in comparison with the convex regular aesthetic abutments, F3.

Many studies, including reviews, have used various different types of surface modifications; it should be noted that the vast majority were performed in vitro or on animals and that, therefore, their results cannot be extrapolated to humans. The results of the in vitro studies show great cell proliferation and adhesion on surfaces treated with various techniques, confirming that surface roughness provides better properties. However, human trials are difficult to carry out due to both ethical concerns and the need to sample peri-implant gingival tissue from the abutment, in addition to the fact that, when carrying out these studies, the samples obtained are relatively small and easily lost [64,65].

Schwarz et al. [55] showed that, according to the histological analysis of the samples obtained from their studies on animals and humans, on the treated surfaces, the distribution and position of the peri-implant connective tissue fibers was perpendicular to the abutment, whereas epithelial connections were more coronal relative to the implant shoulder and thus the peri-implant bone level was higher compared to the untreated abutments. In our study, the difference between the two types of abutments was not significant, favoring the smooth abutments [66]. These results may be attributed to differences in their topography.

Other studies have shown that patients with rough wide neck implants showed less marginal bone loss and less probing depth, compared with rough narrow neck implants placed in the molar-premolar region [67].

On the importance of hydrophilicity, Kim et al., 2015, found that hydrophilic surfaces have a higher probability of cell adhesion compared to anodized surfaces [68], while Xing et al., 2014, in their study on a titanium surface treated with organic acids, obtained a surface with lower hydrophilicity and a greater proliferation of osteoblasts [52,69].

Finally, having reviewed the current situation on the subject of the effects of abutment shape and surface treatment on dental implants, a higher percentage of in vivo studies on humans are needed, since there are many factors which change and affect the results of the variables under examination.

A limitation of our study was the low volume of oral soft tissue of the patients, which prevented a more exhaustive analysis of the abutments, since in most cases the treated patients presented severe maxillary atrophy and consequently a reduced gingival volume.

## 5. Conclusions

Considering the effect of abutment morphology and surface treatment on dental implants, and based on the results of our study on four types of titanium abutments consisting of two shapes, regular (convex) and slim (concave), with two surface treatment techniques, anodized (A) and smooth machined (M) without any type of treatment, we conclude that the slim machined (SM) abutments gave significantly better results compared to the other groups studied (SA, RM and RA) in relation to the variables studied (biological width, dimensions and density of connective and epithelial attachment with infiltrations of inflammatory cells in the area). These results were obtained in the short term (8 weeks), which highlights the need for more long-term studies with a larger sample size, adopting new surface treatment techniques with different distributions along the abutment, and based on the latest research cited.

In conclusion, in addition to the above, more research is necessary on the treatment of the surface of prosthetic abutments, considering the properties of the treated surface on the adhesion of the biofilm and facilitating the good maintenance of hygiene in the area, perpetuating the health of the soft tissue implant that is a key piece in prolonging the life of the implant system.

## Figures and Tables

**Table 1:** Description of the sociodemographic and clinical characteristics of the edentulous patients participating in the study (*n* = 10).

GENDER	N	%
Man	7	70.0
Women	3	30.0
	MEAN	SD
AGE	65.9	10.7
Age range	N	%
<65 years old	24	60.0
≥65 years old	16	40.0
SMOKER	N	%
No	6	60.0
Yes	4	40.0

**Table 2:** Comparison by ANOVA of the effect of both abutment design (parallel vs. convergent) and the surface type (anodized vs. machined) on the gingival tissue strata.

	ABUTMENT DESIGN				SURFACE TYPE			
	Aesthetic (Parallel Walls)		Slim (Convergent Walls)		Anodized		Machined	
TISULAR STRATA	Mean	SD	Mean	SD	Mean	SD	Mean	SD
Biological width(mm)	3.9	1.7	3.6	0.9	3.8	1.4	3.7	1.3
Epithelial sulcus depth(mm) *	0.8 ^a^	0.6	0.4 ^b^	0.3	0.6 ^a^	0.4	0.6 ^a^	0.6
Epithelium length (mm)	2.0	1.1	1.6	0.9	2.0	1.1	1.5	0.8
Connective tissue thickness(mm)	2.0	1.4	2.2	0.8	1.8	0.8	2.3	1.3

* Significant difference between groups after ANOVA. ^a,b^ Distinct uppercase letters indicate the subgroups are significantly different after post hoc Bonferroni corrections.

**Table 3:** Comparison by 2 × 2 chi-square tests of the effect of either abutment design (parallel vs. convergent) or the surface type (anodized vs. machined) on the density and vascularization of the gingival tissues.

HISTOLOGICAL FINDINGS	ABUTMENT DESIGN				SURFACE TYPE			
	AESTHETIC (PARALLEL WALLS)		SLIM (CONVERGENT WALLS)		ANODIZED		MACHINED	
	N	Percentage	N	Percentage	N	Percentage	N	Percentage
Low density	2	25.0%	2	14.3%	1	7.1%	3	37.5%
High density	6	75.0%	12	85.7%	13	92.9%	5	62.5%
Chi^2^ (*p*-value)	Chi^2^ = 0.39 (*p* = 0.53)				Chi^2^ = 3.15 (*p* = 0.08)			
Low vascularization	7	87.5%	9	64.3%	10	71.4%	6	75.0%
High vascularization	2	12.5%	5	35.7%	4	28.6%	2	25.0%
Chi^2^ (*p*-value)	Chi^2^ = 1.38 (*p* = 0.24)				Chi^2^ = 0.03 (*p* = 0.86)			

**Table 4:** Comparison by 2 × 2 chi-square tests of the effect of either abutment design (parallel vs. convergent) or the surface type (anodized vs. machined) on histological inflammation of the peri-implant soft tissues.

Grade of Inflammation *	ABUTMENT DESIGN				SURFACE TYPE			
	AESTHETIC (PARALLEL WALLS)		SLIM (CONVERGENT WALLS)		ANODIZED		MACHINED	
	N	Percentage	N	Percentage	N	Percentage	N	Percentage
Low inflammation	5	62.5%	11	78.6%	11	78.6%	5	62.5%
High inflammation	3	37.5%	3	21.4%	3	21.4%	3	37.5%
Chi^2^ (*p*-value)	Chi^2^ = 0.66 (*p* = 0.42)				Chi^2^ = 0.66 (*p* = 0.42)			

* The grade of inflammation was obtained by counting inflammatory cells during histomorphometric analysis.

**Table 5:** ISQ values.

	All (*n* = 40)		SM (*n* = 10)		SA (*n* = 10)		RM (*n* = 10)		RA (*n* = 10)	
	x	SD	x	SD	x	SD	x	SD	x	SD
beginning	50.9	13.3	50.8	10.8	48.2	17	46.9	16	57.7	5.9
1 Month	45.3	12.0	43.8	11.3	42.4	12.8	43.9	12.8	51.0	10.7
2 Month	45.4	12.0	44.7	10.7	46.1	13.7	43.1	15.0	47.8	9.2

**Table 6:** Distance from abutment shoulder to bone.

	All (*n* = 40)		SM (*n* = 10)		SA (*n* = 10)		RM (*n* = 10)		RA (*n* = 10)	
	x	SD	x	SD	x	SD	x	SD	x	SD
Beginning	3.2	1.3	3.6	1.05	3	1.05	2.7	1.05	3.5	1.8
1 Month	2.8	0.88	2.9	0.87	2.8	0.78	2.9	0.99	2.6	0.96
2 Month	3.05	0.71	2.7	0.48	3.2	0.91	3.2	0.42	3.1	0.87

**Table 7:** Attached keratinized gingival.

	All (*n* = 40)		SM (*n* = 10)		SA (*n* = 10)		RM (*n* = 10)		RA (*n* = 10)	
	x	SD	x	SD	x	SD	x	SD	x	SD
Beginning	5.03	3.1	4.9	3.7	4.8	2.9	5.2	1.6	5.2	3.9
1 Month	4.95	2.68	5.4	2.31	4.6	2.6	4.4	2.7	5.4	3.1
2 Month	4.53	2.85	5.2	3.6	4.3	2.3	3.8	2.2	4.8	3.2

**Table 8:** Height of peri-implant sulcus.

	All (*n* = 40)		SM (*n* = 10)		SA (*n* = 10)		RM (*n* = 10)		RA (*n* = 10)	
	x	SD	x	SD	x	SD	x	SD	x	SD
Beginning	1.17	1.6	0.8	1.76	1.35	1.29	1.55	1.46	1	2.21
1 Month	2.3	2.1	2.0	2.7	2.9	1.9	2.3	1.8	1.9	1.9
2 Month	2.0	1.5	1.5	1.5	2.1	1.3	1.5	1.4	3	1.6

## Data Availability

Not applicable.
